# Clinical diagnosis and etiology of patients with *Chlamydia psittaci* pneumonia based on metagenomic next-generation sequencing

**DOI:** 10.3389/fcimb.2022.1006117

**Published:** 2022-10-13

**Authors:** Yueming Liang, Tingyan Dong, Minjing Li, Peifang Zhang, Xiaoqun Wei, Haitao Chen, Yongsi Wang, Xinglin Gao

**Affiliations:** ^1^ The Second School of Clinical Medicine, Southern Medical University, Guangzhou, China; ^2^ Department of Geriatric Respiratory Medicine, Guangdong Provincial People’s Hospital, Guangdong Academy of Medical Sciences, Guangdong Provincial Geriatrics Institute, Guangzhou, China; ^3^ Integrated Diagnostic Centre for Infectious Diseases, Guangzhou Huayin Medical Laboratory Center, Guangzhou, China; ^4^ The School of Medicine, Nanjing University, Nanjing, China; ^5^ Department of Respiratory and Critical Care Medicine, The First People’s Hospital of Foshan, Foshan, China

**Keywords:** *C. psittaci* pneumonia, clinical diagnosis, etiological analysis, metagenomic next generation sequencing, coinfection

## Abstract

The incidence of severe *Chlamydia psittaci* (*C. psittaci*) pneumonia and coinfections is increasing. Early detection of this condition is needed to prevent negative outcomes, along with detailed descriptions of its associated clinical characteristics. Our study contributes by undertaking etiological analysis of patients with *C*. *psittaci* pneumonia based on metagenomic next-generation sequencing (mNGS). A retrospective analysis of 30 patients with *C. psittaci* pneumonia was undertaken and confirmed by mNGS or polymerase chain reaction (PCR). Clinical manifestations of the severe and non-severe *C. psittaci* pneumonia groups were compared for clinical reference. Etiological analyses were also performed to comprehensively understand pathogeny and coinfection with other respiratory pathogens in *C. psittaci* patients. The absolute value of lymphocytes (LYM) in the severe group was lower than in the non-severe group. At the same time, neutrophil-to-lymphocyte ratio (NLR), procalcitonin (PCT), alanine aminotransferase (ALT), D-II polymer, brain natriuretic peptide (BNP), myoglobin (MYO), and cardiac troponin I (cTnI) were significantly higher (*P* < 0.05) in the severe group. mNGS has a broader pathogen spectrum and can more sensitively detect *C. psittaci* and other low-abundance pathogens with a higher positive detection rate (100%, 13/13 vs. 46%, 6/13, *P <*0.05) than conventional culture methods. mNGS detected the following dominant species associated with *C. psittaci* in patients: bacteria (53.2%, 39% gram-positive, 61% gram-negative), fungi (12.9%), and viruses (33.9%). A total of 73.3% (11/15) of patients had suspected coinfections, with a coinfection rate of 91.7% (11/12) in the severe group. No coinfection or death occurred in the non-severe group. Prognosis in the severe group was poor, with a mortality rate of 27.3% (3/11) for patients with coinfection. Eight of 11 patients with coinfections (72.7%) recovered. In conclusion, the clinical symptoms of severe *C. psittaci* pneumonia manifested as abnormal inflammatory indicators, impaired liver function, myocardial injury, coagulation, and relatively low immune responses. The higher proportion of patients with coinfections in our study supports the use of mNGS for comprehensive early detection of respiratory infections in patients with *C. psittaci* pneumonia. Simultaneous early identification of coinfections would further improve the clinical treatment of these patients.

## Introduction

*Chlamydia psittaci* is an intracellular gram-negative bacterium that commonly infects birds such as parrots, wild pigeons, chickens, and ducks (Gu et al., 2020). It is more pathogenic and reproduces faster than other chlamydial species, causing more severe inflammatory responses and leading to higher mortality (Pendleton et al., 2017). *C. psittaci* mainly infects humans who inhale dust containing respiratory secretions or dried droppings from infected birds (Shaw et al., 2019). Zhang et al. (2022) report the human-to-human transmission of *C. psittaci* in China, but fewer than 5% of *C. psittaci* pneumonia cases were community-acquired (Petrovay and Balla, 2008; de Gier et al., 2018). *C. psittaci* pneumonia has been widely reported worldwide, including in China, the US, Europe, and Australia (Cheng et al., 2013; Branley et al., 2014; Shaw et al., 2019; Shi et al., 2021). Infection in humans can cause serious diseases, including pneumonia, acute respiratory distress syndrome (ARDS), sepsis, septic shock, and multiple organ system failures, and death (Kovacova et al., 2007; Hogerwerf et al., 2017; Wang et al., 2021). Death is more likely to occur among adults than children, although *C. psittaci* pneumonia is rarely fatal (< 1%) (Charles et al., 2008; Balsamo et al., 2017; Hogerwerf et al., 2017). Typically, the disease occurs after an incubation period of 5 to 14 days (Heddema et al., 2006). It mainly infects the lungs, liver, spleen, and meninges, and typical clinical manifestations include fever, chills, headache, dry cough, gastrointestinal problems, severe pneumonia, endocarditis, jaundice, and nervous system complications (Rybarczyk et al., 2020; Shen et al., 2021).

Early diagnosis of *C. psittaci* pneumonia is challenging because the disease is characterized by non-specific symptoms, and few diagnostic tests are available (Schlaberg et al., 2017). Culturing is not an ideal method for *C. psittaci* pneumonia identification–it is time-consuming and complex, with low sensitivity. The diagnostic efficiency of serological assay and PCR methods cannot also be guaranteed. Therefore, most human *C. psittaci* infections remain undetected globally, and the incidence and disease burden might be greatly underestimated. Metagenomic next-generation sequencing (mNGS) is a relatively new assay for the rapid and precise detection of pathogenic microorganisms (viral, bacterial, fungal, or parasitic) without bias through sequencing, which may assist in *C. psittaci* pneumonia diagnosis (Langelier et al., 2018). The approach has been gradually applied in clinical practice in recent years, particularly for severe pneumonia in intensive-care unit (ICU) settings (Yang et al., 2021), and has been demonstrated as an effective method for establishing clinical diagnoses.

*Streptococcus pneumoniae* and *Haemophilus influenzae* are the most common pathogens to cause community-acquired pneumonia (Brooks and Mias, 2018). However, most atypical cases are caused by mycoplasma infections, and *C. psittaci* cases have not been widely reported (Hughes et al., 1997; Mair-Jenkins et al., 2018). We observed that *C. psittaci* causes atypical pneumonia more frequently, and patients usually develop severe pneumonia in such cases. As guidelines recommend (Cao et al., 2018), pathogenic examination, including blood and sputum cultures, should be performed before using specific antibiotics. Broad-spectrum antibiotics are typically prescribed for patients with severe pneumonia to cover all possible pathogens before the results of empirical examinations are available. If the clinical characteristics of *C. psittaci* pneumonia could be readily identified and the tests are performed early, there would be no delay in applying targeted therapy. Emerging evidence indicates that *C. psittaci*-infected individuals are at increased risk for coinfections, although the process of concomitant infection by other respiratory pathogens and *C. psittaci* is still unclear. A better understanding of the prevalence of coinfection in patients with *C. psittaci* pneumonia and the profile of pathogens would contribute to more effective patient management and antibiotic stewardship during the current pandemic. Our study focused on the *C. psittaci* pneumonia cases in one medical center, aiming to summarize and compare the clinical characteristics of patients with severe and non-severe *C. psittaci* pneumonia. We also analyzed the potential value of mNGS in detecting *C. psittaci* to improve being alerted about this and other treatable pathogens and increase doctors’ awareness of *C. psittaci* pneumonia.

## Materials and methods

### Participants and study design

A total of 30 patients were diagnosed with *C. psittaci* pneumonia. Five patients’ diagnoses were confirmed by real-time fluorescent quantitative PCR (RT-PCR) and 25 by mNGS in the Foshan First People’s Hospital from January 2020 to December 2021. Following the criteria for severe pneumonia diagnosis (Mandell et al., 2007), 23 patients were identified as having severe pneumonia and seven with non-severe pneumonia. Basic information including age, gender, underlying diseases, history of exposure to birds or poultry, and symptoms was extracted from their medical records. Chest computed tomography (CT) and chest X-ray results were recorded during hospitalization (including the time of diagnosis and treatment) and follow-up care. Clinical indicators tested within 24 h of admission were recorded, such as blood routine, C-reactive protein (CRP), procalcitonin (PCT), electrolytes, brain natriuretic peptide (BNP), myocardial enzymes, liver and kidney function, D-dimer, and oxygenation index (PO2/FiO2). Treatment strategies and relevant changes during follow-up were also noted.

**Figure 1:**
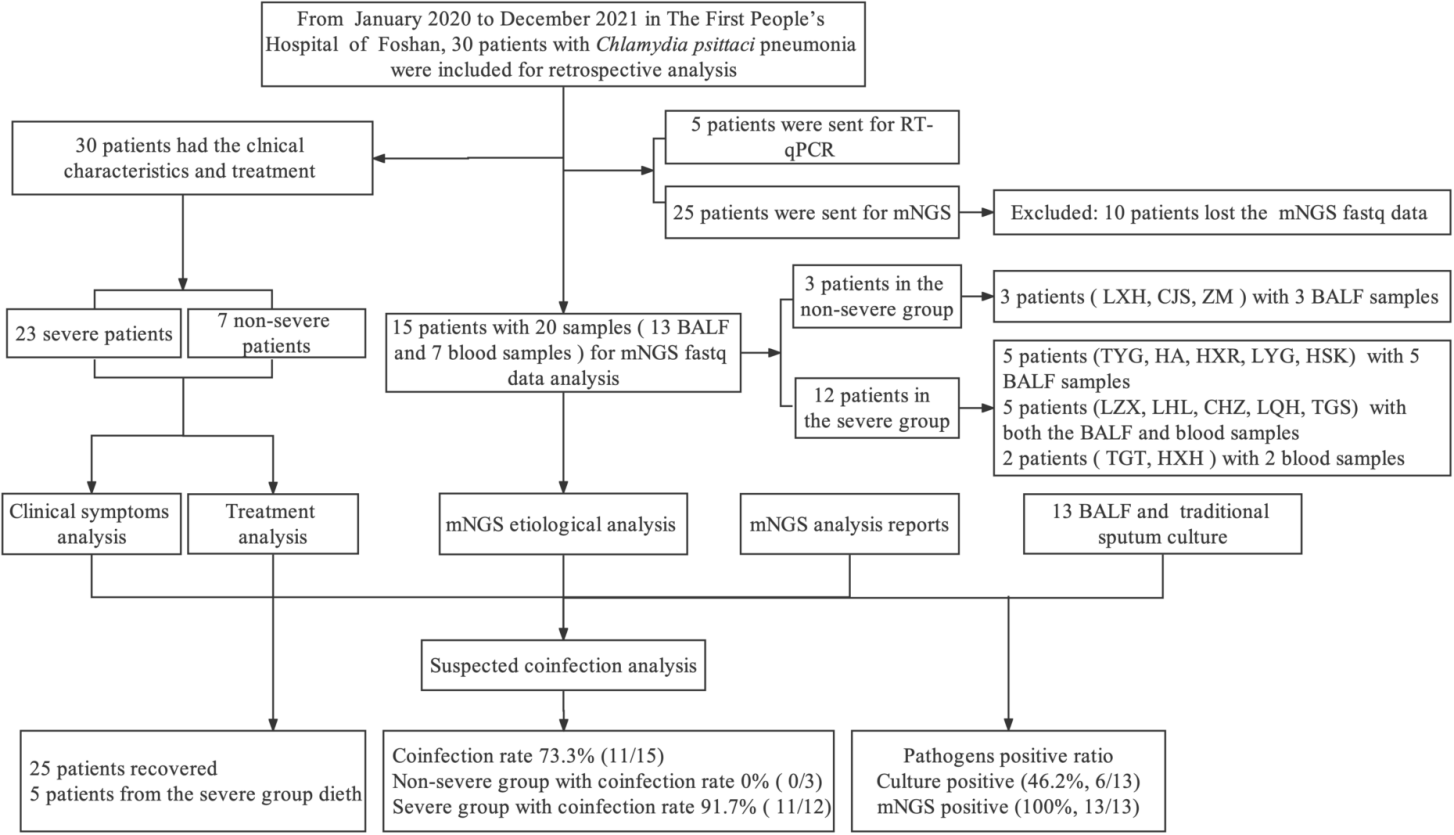
The research route for this study design. BALF: bronchoalveolar lavage fluid.

Of the 25 patients diagnosed with *C. psittaci* infection by mNGS, 10 patients whose mNGS fastq data were lost were excluded. Further etiological analysis and suspected coinfection analysis were performed only for 15 patients. Specifically, we analyzed 20 samples [13 bronchoalveolar lavage fluid (BALF) and seven blood samples] from 15 patients. Three patients (LXH, CJS, and ZMY) from the non-severe group provided three BALF samples, and 12 patients from the severe group provided 10 BALF and seven blood samples. Five patients (TYG, HA, HXR, LYG, and HSK) individually provided five BALF samples, and a further five patients (LZX, LHL, CHZ, LQH, and TGS) had both BALF and blood samples, while two patients (TGT and HXH) provided two blood samples (**Figure 1**). The results of the mNGS report and sputum culture were also analyzed to determine the positive detection ratio. This study was approved by the Ethics Committee of the First People’s Hospital of Foshan (K (2019)-3).

### Diagnostic criteria for severe pneumonia

The patients were divided into severe and non-severe groups based on clinical condition. The diagnostic criteria for severe pneumonia were based on the eighth edition of Internal Medicine, the Infectious Diseases Association of America/American Thoracic Society (IDSA/ATS), and the Guidelines for the Diagnosis and Treatment of Community-Acquired Pneumonia in Chinese Adults (Mandell et al., 2007). Patients diagnosed with severe pneumonia had to fulfill at least one major criteria, or at least three secondary criteria. The major criteria were that mechanical ventilation with tracheal intubation was considered necessary and septic shock required vasoactive drug therapy after active fluid resuscitation. Secondary criteria included (1) respiratory rate ≥ 30 breaths/min; (2) PaO2/FiO2 ratio ≤ 250; (3) multilobar infiltrates; (4) confusion/disorientation; (5) blood urea nitrogen ≥ 7.14 mmol/L; and (6) systolic blood pressure < 90 mm Hg, hypotension requiring aggressive fluid resuscitation.

### mNGS diagnostic method

Clinical samples were collected by following the standards of aseptic processing procedures: (1) A 1.5–3 mL of the BALF sample or 5 mL of blood sample was collected from each patient. Nucleic acid extraction took place using a TIANamp Micro DNA Kit (DP316, Tiangen Biotech Co., Beijing, China). (2) DNA libraries were constructed using the VAHTS Universal Plus DNA Library Prep Kit for Illumina (ND617-C2, Vazyme Biotech Co., Nanjing, China). Agilent 2100 was used for quality control of the DNA libraries. Qualified libraries were sequenced by the Illumina 550 platform. (3) High-quality sequencing data were generated, followed by computational subtraction of human host sequences mapping to the human reference genome (hg19) using Kraken2 2.1.2 and Burrows-Wheeler Alignment (BWA). Low-quality and short reads (length < 50 bp) were removed by fastp 0.20.1. The remaining data were aligned to the Pathogenic Microbial Genome Databases, including bacteria, viruses, fungi, and parasites. The classification reference databases were downloaded from National Center of Biotechnology Information (NCBI) (ftp://ftp.ncbi.nlm.nih.gov/genomes/) and the mapped data were processed for advanced analysis.

### Statistical analysis

SPSS 21.0 (IBM Corp.: Armonk, NY, US) was used for clinical data analysis. Continuous measurement data following normal distribution were shown as mean ± standard deviation (x ± s), and *t*-tests were used to compare groups. Non-normal distribution was shown by median (interquartile range), and Mann-Whitney U tests were used when two groups were being compared. *P* < 0.05 was considered statistically significant.

Analysis of the mNGS results included several stages. At least two reads were mapped to the pathogens, except for specific pathogens such as *Bacillus anthracis, Orientia tsutsugamushi, Chlamydia pneumoniae, Brucella, Cryptococcus neoformans, Coronavirus, Human Immunodeficiency Virus (HIV)*, and *Toxoplasma*, whose relative abundances would be surpassed (**Formula**) (Zhang et al., 2020). The preliminary data contained the relative abundance of microbes detected by mNGS and each microbe’s threshold for further test validation as follows: (1) Pathogens with the highest absolute abundance in their genus (Top 5) and (2) Species with relative abundance ≥ 5% for bacteria, ≥ 50% for fungi, and ≥ 10% for viruses, were considered dominant species.


$$Absolute\;Abundance=\frac{Reads\;Number}{Whole\;Genome\;Size}\times 10^{6}$$

$$Relative\;Abundance=\frac{Absolute\;Abundance\;}{\sum_{This\;sample}Absolute\;Abundance\;}\times \;100\%$$

## Results

### Clinical characteristics of severe and non-severe groups

In total, 30 patients (16 women and 14 men) with *C. psittaci* pneumonia were studied. Five patients were confirmed as having *C. psittaci* pneumonia by RT-qPCR. They were from the same village and had been exposed to the same batch of livestock (chickens) by accident. Two-thirds (20/30) of the patients had no clear history of exposure to birds or poultry, and up to 78.3% (18/23) of the patients with severe *C. psittaci* pneumonia reported no history of contact with animals (**Table 1**). The main risk factors for *C. psittaci* pneumonia are exposure to chickens or human-to-human transmission. The non-severe group comprised seven patients aged 38–71 years, 85.7% (6/7) were women and with an average age of 56.00 ± 11.11 years. In the severe group, 56.5% (13/23) were men and 43.5% (10/23) were women, aged from 43–85 years, with an average age of 59.74 ± 12.11 years. Approximately half (47.8%, 11/23) of the patients in the severe group had complications. One patient had breast cancer in the non-severe group, compared with 30.4% (7/23) of patients with underlying conditions in the severe group. Specifically, one patient had a history of chronic obstructive pulmonary disease; three patients had hypertension; one patient had diabetes and had undergone a splenectomy due to trauma; one patient had a history of gout, and another had a history of hepatitis C cirrhosis and drug use. None of the patients had used any immunosuppressive agents. In terms of clinical symptoms, all 30 patients had a remittent fever higher than 39°C, accompanied by weakness, coughing, difficult breathing, and sputum production. Pulmonary signs, such as rhonchi and wet or dry rales in both lungs, were observed in 83% (25/30) of patients. Compared with the non-severe group’s mild clinical manifestations, the clinical symptoms of patients with severe *C. psittaci* pneumonia mainly manifested as difficult breathing (100%, *P* < 0.05), abnormal liver function (100%, *P* < 0.001), myocardial damage (82.6%, *P* < 0.001), and bilateral pneumonia (100%, *P* < 0.001), and assisted ventilation (69.6%, *P* < 0.01) (**Table 1**).

**Table 1:** Comparison of basic clinical data for severe and non-severe groups.

Characteristics	Non-severe group (*n *= 7)	Severe group (*n *= 23)	*P-*value	All patients (*N *= 30)
Demographics				
Age (x ± s, year)	56.00 ± 11.11	59.74 ± 12.11	0.622	58.86 ± 17.23
Men (n, %)	1 (14.3)	13 (56.5)	0.086	14 (46.7)
Women (n, %)	6 (85.7)	10 (43.5)	——	16 (53.3)
Contact history (n, %)	5 (71.4)	5 (21.7)*	0.026	10 (33.3)
Complication	1 (14.3)	11 (47.8)	0.193	12 (52.2)
Survival	7 (100.0)	18 (78.4)	0.304	25 (83.3)
Underlying disease	1 (14.3)	7 (30.4)	0.638	22 (73.3)
Immunosuppressive agents	0 (0.0)	0 (0.0)	——	0 (0.0)
Clinical symptoms				
Fever	7 (100.0)	23 (100.0)	——	30 (100.0)
Weakness	6 (85.7)	23 (100.0)	0.233	29 (96.7)
Intrapulmonary (n, %)				
Cough	7 (100.0)	23 (100.0)	——	30 (100.0)
Breathing difficulty	5 (71.4)	23 (100.0)*	0.048	28 (93.3)
Extrapulmonary (n, %)				
Disturbance of consciousness	0 (0.0)	1 (4.35)	1.000	1 (3.3)
Abnormal liver function	2 (28.6)	23 (100.0)***	0.0001	25 (83.3)
Abnormal renal function	1 (14.3)	3 (13.0)	1.000	4 (13.3)
Myocardial damage	2 (28.6)	19 (82.6)***	0.0009	21 (70.0)
Electrolyte disorder (hyponatremia)	3 (42.9)	19 (82.6)	0.059	22 (73.3)
Chest CT or X-ray (n, %)				
Bilateral pneumonia	2 (28.6)	23 (100.0)***	0.0001	25 (83.3)
Direct invasion of the pleura	1 (14.3)	5 (21.7)	1.000	6 (20.0)
Assisted ventilation	0 (0.0)	16 (69.6)**	0.002	16 (53.3)

*P<0.05, **P < 0.01, ***P < 0.001.

### Laboratory testing of severe and non-severe groups

**Figure 2:**
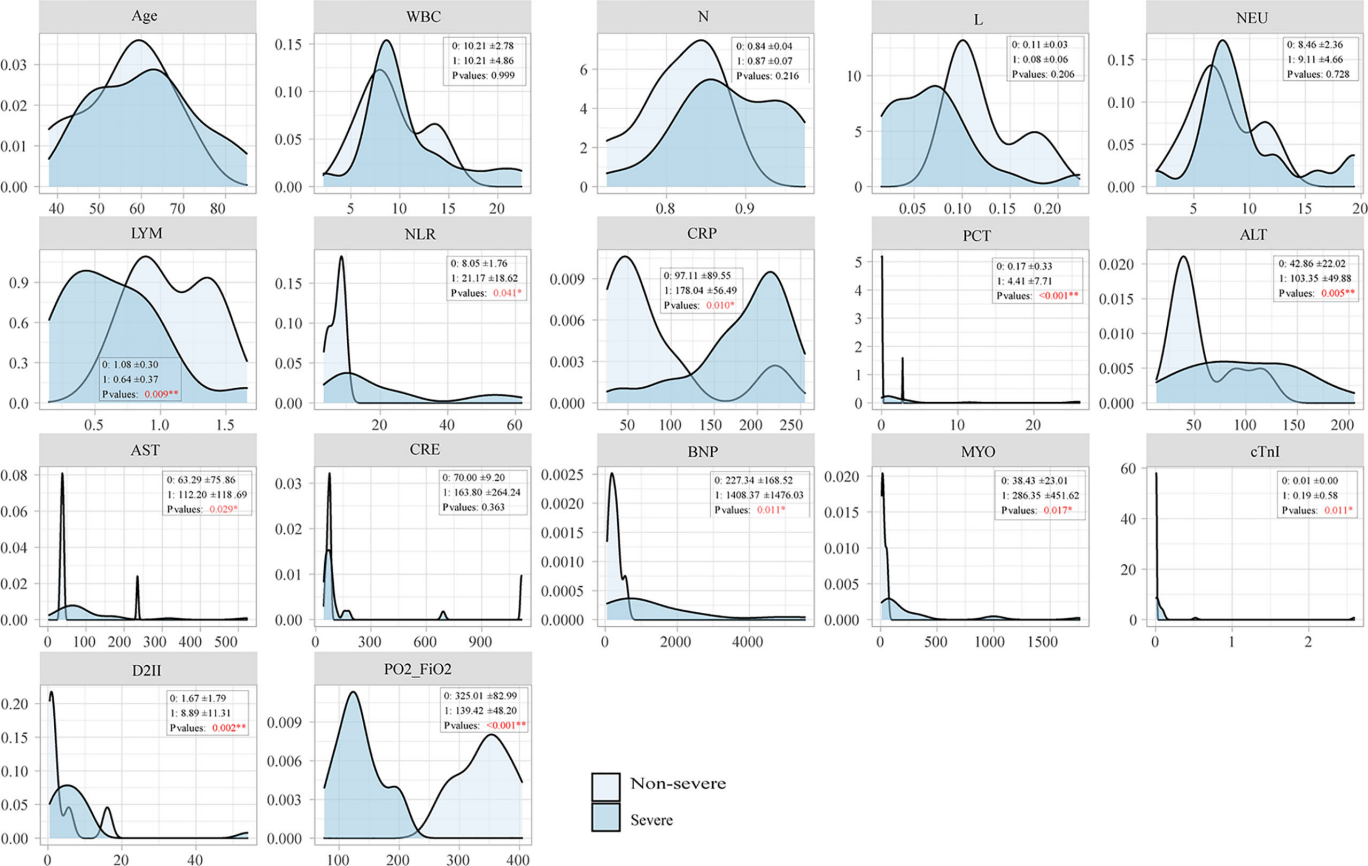
Comparison of laboratory tests between the non-severe and severe groups.

Laboratory indicators showed significant differences between the severe and non-severe groups. The oxygenation index ranged from 85.85 to 202 in the severe group. The absolute value of LYM in the severe group was lower than for the non-severe group, and NLR, PCT, ALT, D-II polymer, BNP, MYO, and cTnI were significantly higher (*P* < 0.05) in the severe group. There were no significant differences in age, comorbidities, white blood cells (WBC), absolute neutrophils (NEU), renal function, and hyponatremia between the two groups, as shown in **Figure 2**.

### Technical investigations

**Figure 3:**
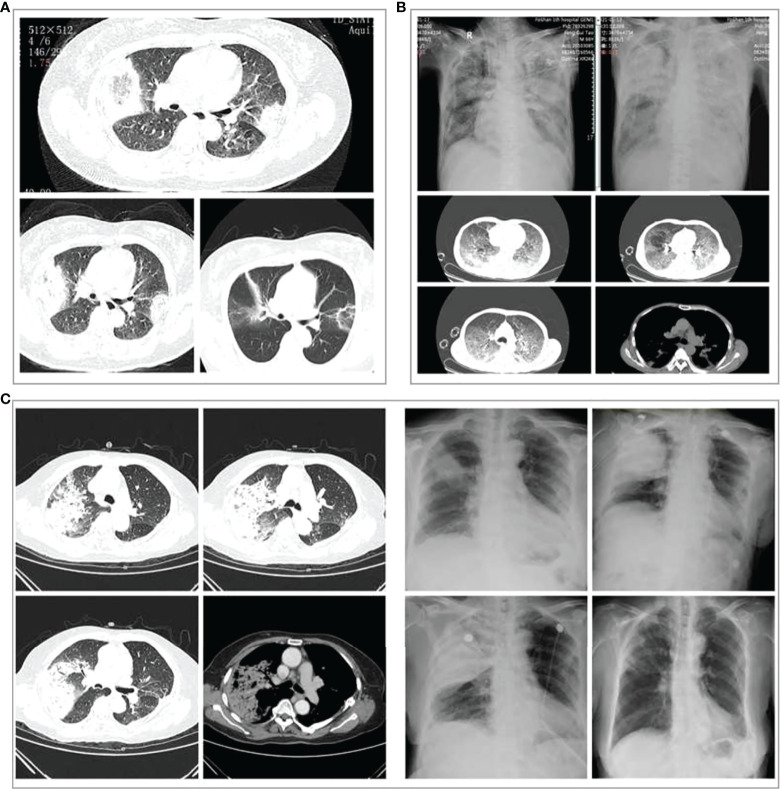
Chest imaging changes in three patients with severe *Chlamydia psittaci* pneumonia during hospitalization. **(A)** Chest computed tomography (CT) scans of a 60‐year‐old woman with severe *C. psittaci* pneumonia. CT scan (3 days after treatment) showed both lungs with reversed halo signs (RHS). **(B)** Chest X-ray and CT of a 66‐year‐old man with severe *C. psittaci* pneumonia. Chest X-ray showed a large consolidation-like shadow in the upper right lung. Chest CT showed diffuse interstitial changes in both lungs. **(C)** Chest X-ray and CT of a 74‐year‐old woman with severe psittacosis pneumonia. Chest CT showed massive consolidation in the upper lobe of the right lung. Chest X-ray (dynamic views) showed that the consolidation increased rapidly in the upper lobe of the right lung within a short period, and the absorption improved after treatment, with only a few fibrous foci remaining.

Chest CT scans showed pulmonary inflammatory infiltrates with interstitial inflammation, which could be unilateral or bilateral. Patients in the severe group had more bilateral lung lesions than the non-severe group (**Table 1**). Rapidly progressive pleural effusion (**Figure 3B**) or multilobar consolidation (**Figure 3C**), diffuse interstitial changes in both lungs, and ARDS manifestations were observed in the severe group. One patient presented with an anti-halation sign (**Figure 3A**). A small fibrous cord-like exudation could be observed on chest CT during the absorption period, as shown in **Figure 3C**. Most patients from the non-severe group showed lobular-centered shadows.

### Etiological diagnostic performance of mNGS in *C. psittaci* patients

**Figure 4:**
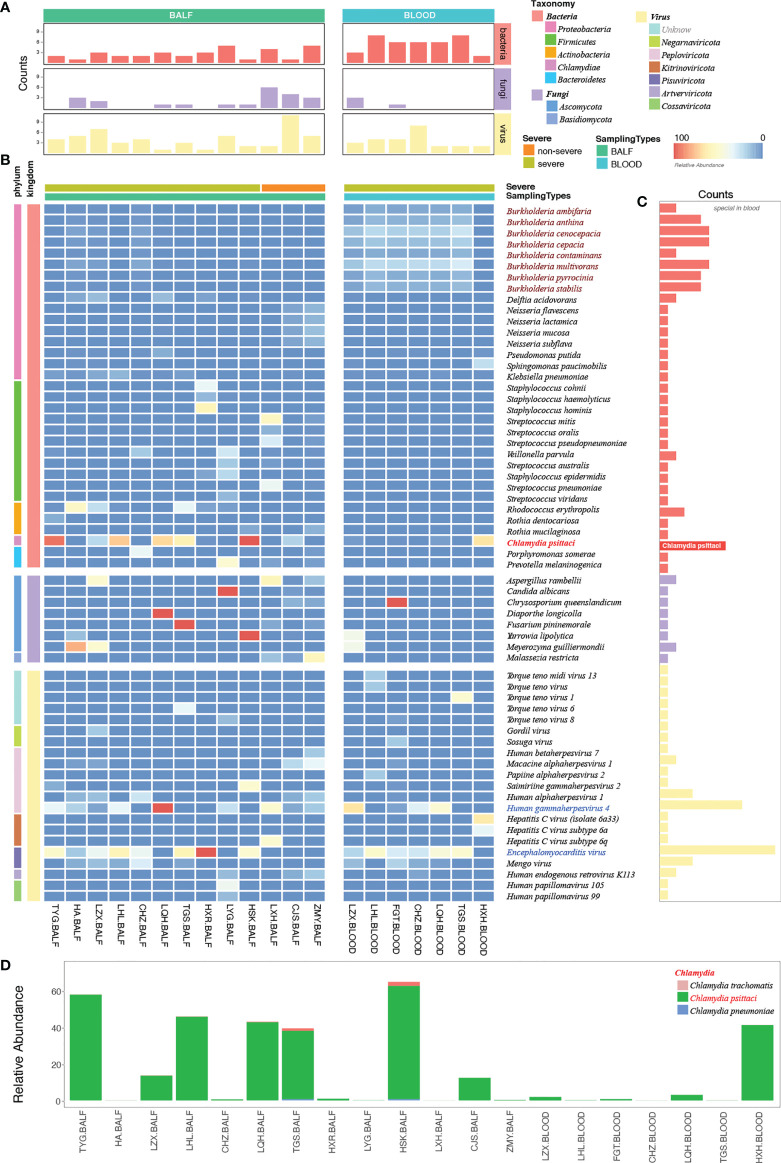
Etiological analysis of the bronchoalveolar lavage fluid (BALF) and blood samples from patients with *Chlamydia psittaci* pneumonia by metagenomic next-generation sequencing (mNGS). **(A)** Numbers of dominant species, including bacteria, fungi, and viruses in each sample. **(B)** The heatmap depicts the relative abundances of dominant species assigned to bacteria, fungi, and viruses (y-axis) across the 20 samples analyzed (x-axis). The heatmap colors represent the relative abundances of the microbial species assignments within each sample. Colors of the squares shifted towards red to indicate higher abundance. **(C)** Numbers of each species in the 20 samples. **(D)** The relative abundance of *Chlamydia* in each sample. Square colors represent the different species.

mNGS has a broad pathogen spectrum. Twenty samples (13 BALF and seven blood samples) from 15 patients showed more species of bacteria than viruses and fungi (**Figure 4A**). More species were detected in BALF than in the blood samples (**Supplementary Figure 1**). mNGS identified a total of 62 dominant species (relative abundance ≥ 5% in bacteria, ≥ 50% in fungi, ≥ 10% in viruses) comprising 33 bacteria, eight fungi, and 21 viruses from the 20 samples (**Figure 4B**; **Supplementary Table 1**). Bacteria belonging to the five phyla, namely, *Proteobacteria, Firmicutes, Actinobacteria, Chlamydiae*, and *Bacteroidetes*, were the dominant species in the heatmap. *Burkholderia*, an order of *Proteobacteria*, had a higher relative abundance than *C. psittaci* and occurred in almost all the blood samples of patients with severe pneumonia, indicating a considerably mixed infection (**Figures 4B, C**, and **Supplementary Figure 2**).

The high sensitivity of mNGS led to the detection of *C. psittaci*, although the relative abundance of *C. psittaci* in the blood was critically low. Metagenomes are based on genome and transcriptome sequencing, which can lead to false detection because of high similarity in homologous species (Rhie et al., 2021). Two other homologous species, *Chlamydia trachomatis* and *Chlamydia pneumoniae*, were also detected by mNGS (**Figure 4D**) and were suspected to be false detections, considering their much lower relative abundance. Standards and methods to improve the reliability of metagenomic testing need to be further developed.

The dominant species were the fungi *Aspergillus rambellii* and *Meyerozyma gullermondii* (**Figure 4B**). However, we detected many fungi based on a simple relative abundance filter of > 5% because the relative abundance was calculated based on 100% relative indexing of bacteria/fungi/parasites/viruses. This method can improve the high sensitivity of detection of different species. In this study, 21 strains of viruses were detected by mNGS (**Figure 4B**). The Encephalomyocarditis virus (EMCV) and Human gammaherpesvirus 4 (EBV) were found in most patients’ BALF and blood samples with a higher relative abundance, suggesting that attention should also be paid to viruses mixed with *C. psittaci* infection.

### Coinfection with other pathogens in patients with *C. psittaci* pneumonia based on mNGS, culture, and report

In this study, we identified suspected coinfection with one or more pathogens in patients with *C. psittaci* pneumonia. Information about other relevant pathogens was collected by reviewing their mNGS data based on a relative abundance of ≥ 5% in bacteria, ≥ 50% in fungi, and ≥ 10% in viruses. Overall, 62 dominant species were identified among patients infected with *C. psittaci* (**Supplementary Table 2**). A comprehensive evaluation was performed to assess the suspected coinfection, integrated clinical experience, manifestations, and culture results with the mNGS results. Of the 15 patients, four patients (LXH, CJS, and ZMY in the non-severe group and TYG in the severe group) were assessed as without coinfection, and 11 patients (severe group) were assessed as having suspected coinfection according to their clinical data (**Supplementary Table 3**). The potential pathogens (**Supplementary Table 3**, bold font) in each patient were also evaluated. mNGS and culture results indicated that 73.3% of patients (11/15) had suspected coinfections with a coinfection rate of 91.7% (11/12) in the severe group. Coinfection or death did not occur in the non-severe group. Our results indicate a higher coinfection rate in the severe cases of patients with *C. psittaci* pneumonia. However, this conclusion is tentative due to the limitation of sample volume. Three patients (HA, LYG, and FGT) with coinfection (27.3%, 3/11) died–suggesting a poor prognosis in the severe group while the remainder recovered (**Supplementary Table 3**). Of the five deaths in this study, three (HA, LYG, and FGT) appeared to have been caused by coinfection with *C. psittaci* and other pathogens (HA: Encephalomyocarditis virus; LYG: *Candida albicans*, encephalomyocarditis virus; and FGT: *Chrysosporium queenslandicum*, encephalomyocarditis virus), and the others (LFX: 49 < 59 years, no basic disease and LYC: 68 > 59 years, with basic disease) due to *C. psittaci* infection alone. In particular, LFX died of the inflammatory storm of *C. psittaci* infection, while LYC died of respiratory failure due to *C. psittaci* infection and poor pulmonary function. Deaths caused by *C. psittaci* inflammatory storm and coinfection should receive more attention. The higher proportion of patients with coinfections in our cohort suggests systemic use of antibiotics (**Supplementary Table 4**) in patients with severe *C. psittaci* pneumonia with rapid de-escalation based on respiratory mNGS results.

**Figure 5:**
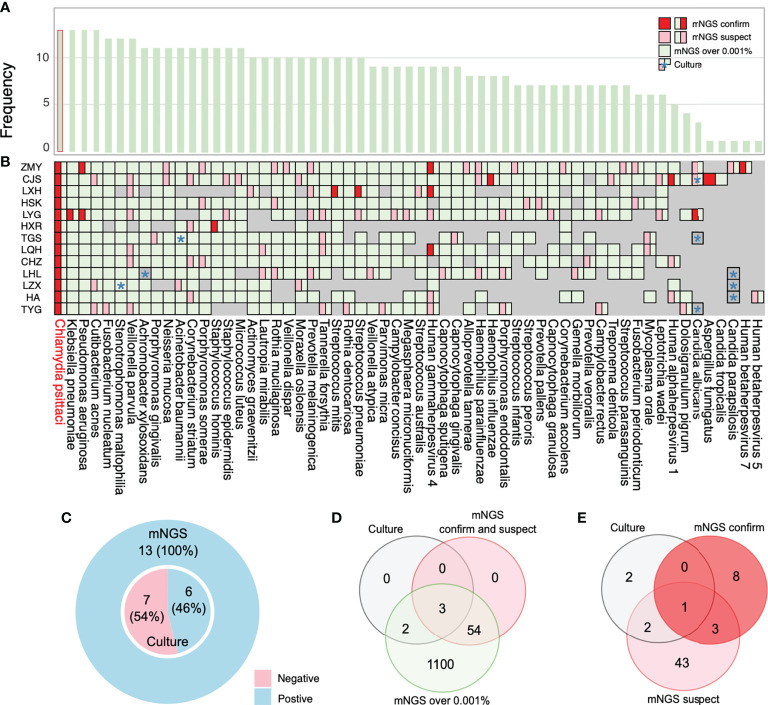
Coinfection analysis and comparisons among the results of metagenomic next-generation sequencing (mNGS) analysis, culture, and reports. **(A)** Frequency of species in 13 bronchoalveolar lavage fluid (BALF) samples. **(B)** Pathogens reported by sputum culture, and mNGS reports and analysis are shown through the chart. Information regarding species in the chart was collected by more than 0.001% relative abundance based on the mNGS analysis results. All pathogens labeled with green are the overall mNGS detection results. The pink and red parts imply that mNGS reports have been suspected and confirmed, respectively. **(C)** The ratio of mNGS-positive patients and conventional culture-positive patients. **(D)** The number of pathogens reported by sputum culture and mNGS-confirmed positive cases and suspected positive cases are shown in the Venn diagram to analyze the consistency ratio between mNGS reports and culture. **(E)** The frequency of pathogens detected by culture and mNGS reports. *: culture results.

This study’s high rate of coinfection could be because mNGS can detect a wide variety of pathogens compared with primary culture and PCR methods. mNGS yields a higher positive detection rate for pathogens compared with the culture method (100%, 13/13 vs. 46%, 6/13) (**Figure 5C**) and tends to be more sensitive to a broad range of pathogens (1,159 vs. 5) (**Figure 5D**; **Supplementary Table 5**). We also compared the mNGS analysis results with the mNGS reports and bacterial culture results (**Figures 5A, B**) and found that the mNGS reports differed from the bacterial culture results. Five pathogens were identified by both the culture and mNGS, but only three pathogens were stated in the company mNGS reports (**Figure 5B**). The mNGS reports identified 57 pathogens (12 confirmed and 45 suspected pathogens) from the samples, but only one from the culture results was consistent with the mNGS reports (**Figure 5E**; **Supplementary Table 6**).

### Treatments

All patients received doxycycline-based antibiotic therapy. In the severe group, 18 patients received moxifloxacin injection combined with oral doxycycline, and tigecycline was administered to two patients. One patient received an injection of moxifloxacin combined with oral doxycycline in the non-severe group. Multiple anti-infective treatments were given to the severe group to mitigate the possibility of cross-infection. One patient in the severe group received combined treatment with extracorporeal membrane oxygenation (ECMO) and 40 mg of methylprednisolone (once a day for 3 days) and finally recovered. In total, 25 patients recovered after treatment. Complete absorption of pulmonary lesions takes 3 to 4 weeks, and some fibrous cord-like lesions may remain afterward. The causes of the five fatalities were cardiogenic pulmonary edema (one patient), ARDS and multiple organ failure (three patients), and respiratory failure (one patient).

## Discussion

This paper contributes novel insights into the etiology and coinfection analysis of *C. psittaci* pneumonia in patients in China, using an mNGS approach. Cases of human-to-human transmission of *C. psittaci* are increasing, and coinfection with *C. psittaci* and other respiratory pathogens places patients at an increased risk. However, the process of concomitant infection is still unclear. Our study systematically analyzed the clinical manifestation of *C. psittaci* pneumonia and pathogen coinfections.

Clinical symptoms and laboratory indices of *C. psittaci* pneumonia in severe and non-severe groups were summarized and shown to be significantly different at a clinical level for diagnosis and treatment. Patients in the severe group had extrapulmonary manifestations resulting in ARDS, sepsis, septic shock, and multiple organ failures. Chest X-ray or CT showed mainly interstitial pneumonia with consolidation and less pleural effusion. In the severe group, only a small amount of fibrous focal exudation remained in the dissipating stage. *C. psittaci* can induce a severe inflammatory storm similar to that caused by the novel coronavirus in cases of COVID-19 (Lei et al., 2021). Laboratory testing manifested low absolute lymphocyte counts, multiple viscera damage, and elevated NLR. NLR is closely related to suppressed immune function, which may more accurately reflect the state of systemic inflammation. Therefore, whether NLR could be used as a predictor of severe disease or death in severe *C. psittaci* pneumonia needs to be further studied. The PCT value in our severe group was higher, indicating a combination of sepsis and mixed infection from multiple bacteria. The mNGS results confirmed the mixed infection findings.

mNGS may determine unknown infection sources and can quantify the relative abundance of pathogens. It has the potential to assist doctors in the diagnosis and treatment of possible mixed infections. In this study, mNGS reported 39% gram-positive and 61% gram-negative bacteria. This is consistent with previous reports of thoracic infections dominated by gram-negative bacteria (Fiore et al., 2019). We found a relative abundance of *Burkholderiales* and lower numbers of *C. psittaci* in blood samples. These patients developed symptoms such as neurological comorbidities, hepatic, renal, and coagulation disorders, and sepsis, which may be caused by the *C. psittaci* and *Burkholderiales* mixed infection. *Burkholderiales* can often be found in contaminated hospital environments, including tap water, thermometers, nebulizers, and catheters (Voronina et al., 2015). Antibiotics for *Burkholderiales* should also be considered during treatment for severe *C. psittaci* pneumonia. EMCV and EBV were also found in abundance in most patients’ BALF and blood samples. EMCV is a small non-enveloped single-strand RNA virus and is the causative agent of myocarditis, encephalitis, and neurological diseases in many mammalian species (Carocci and Bakkali-Kassimi, 2012). EBV infects B cells of the immune system and epithelial cells and causes infectious mononucleosis (Jenson, 2011; Kempkes and Robertson, 2015). Physicians should focus more on these viral infections because patients with *C. psittaci* pneumonia also showed symptoms of central nervous system infection and extremely low immunity.

Coinfections in patients with *C. psittaci* pneumonia may lead to more adverse outcomes and need further investigation. This observational study reported bacterial–viral coinfections in patients with *C. psittaci* infection. The bacterial coinfections were mostly related to *C. psittaci, P. aeruginosa, A. baumannii*, and *K. pneumoniae*. The common concomitant viral pathogens in our cohort were gammaherpesvirus 4 (EBV) and Encephalomyocarditis virus (EMCV) in blood samples and Human alphaherpesvirus 1 in BALF samples. Clinicians should anticipate coinfections and must have a high index of suspicion for coinfections and secondary infections in patients with *C. psittaci* pneumonia. Screening for other respiratory pathogens during the clinical course of severe *C. psittaci* pneumonia is critical for appropriate diagnosis and treatment. Empirical antibiotic treatment should be prescribed for patients with severe *C. psittaci* pneumonia, with rapid de-escalation based on mNGS/culture results.

We analyzed the possible reasons for the inconsistent mNGS report results with the culture results. The overall unique sequence/reads were lower in samples detected by culture while undetected by the mNGS report. For example, mNGS reported higher reads of *A. baumannii* for TGS and *Stenotrophomonas maltophilia* for LZX. However, LHL’s lower reads of *Achromobacter xylosoxidans* were not reported because of the lower extract concentration of fungal nucleic acid (**Figure 5B**; **Supplementary Table 1**). Fungal wall-breaking technology should be considered to improve fungal detection ability. Clinicians should also consider the results of traditional cultures when selecting mNGS and make accurate judgments based on clinical practice. *C. albicans* was cultivated for CJS, TGS, and TYG, but only reported in CJS’s sample by mNGS. We found that CJS’s unique reads were several times higher than those of TGS and TYG. Furthermore, *Candida parapsilosis* was undetected in samples from HA, LZX, and LHL but was detected by mNGS in ZMY’s sample. The unique reads of ZMY’s sample were much higher than those from HA, LZX, and LHL (**Figure 5B**; **Supplementary Table 1**). Therefore, relative detection thresholds for different species are needed because of varying degrees of difficulty for nucleic acid extraction. Some authors have suggested increasing the sequencing throughput (such as 20 M) (Liu et al., 2021) and threshold tests for targeted species, ensuring a high throughput and sequencing stability over time. Generally, we suggest using the mNGS results for early diagnosis (PCR verification, if possible) when mNGS report results are inconsistent with culture results. The mNGS results rapidly provide the most potential pathogens to assist clinicians with diagnosis. Although some positive results may not be the underlying cause of the disease, a list of potential pathogens helps provide useful clues.

In conclusion, mNGS has an overall superior detection rate and broader pathogen spectrum than traditional methods and may be particularly useful for concomitant infections and rare pathogens that are difficult to culture. Nevertheless, our study has some limitations. First, more samples from patients with *C. psittaci* pneumonia must be statistically analyzed for coinfection pathogens. Second, the retrospective study design did not allow for the use of PCR, complement-fixation testing, or micro-immunofluorescence to confirm all the mNGS results. mNGS detection needs to be improved; for instance, authoritative guidance about interpreting mNGS reports is required. Some pathogens with hard cell walls, such as fungi, may reduce the extraction efficiency of nucleic acid, leading to low detection sensitivity (Fredricks et al., 2005; Limon et al., 2017). Even if the sequence number of such pathogens was not high in the detection report, the possibility of being pathogenic should still be considered. Third, it is difficult to discern whether the bacterial infections reported in this study are of a community-acquired or nosocomial origin. Hence, physicians should consider the clinical significance of these pathogens when treating patients with severe *C. psittaci* infection.

## Data availability statement

The data sets presented in this study can be found in online repositories. The names of the repository/repositories and accession number(s) can be found at NCBI, PRJNA865167.

## Ethics statement

The studies was approved by the Ethics Committee of The First People’s Hospital of Foshan. The participants provided their written informed consent to participate in this study.

## Author contributions

YL and TD contributed to interpreting the data, and drafted and revised the article. ML undertook data acquisition. PZ took charge of the manuscript review. HC and YW undertook data analysis. XG was responsible for the conception, design, and critical revisions of the article. All authors contributed to the article and approved the submitted version.

## Funding

This work was supported by the Medical Research Foundation of Guangdong Province in 2020 (#B2020146).

## Acknowledgments

The authors thank all patients and their families who participated in the study. They also thank Hao Zhou, Junting Xie, and Jiawen Zhang for their help with the suggestions.

## Conflict of interest

The authors declare that the research was conducted in the absence of any commercial or financial relationships that could be construed as a potential conflict of interest.

## Publisher’s note

All claims expressed in this article are solely those of the authors and do not necessarily represent those of their affiliated organizations, or those of the publisher, the editors and the reviewers. Any product that may be evaluated in this article, or claim that may be made by its manufacturer, is not guaranteed or endorsed by the publisher.
