# Supplementary material for: Clinical diagnosis and etiology of patients with Chlamydia psittaci pneumonia based on metagenomic next-generation sequencing
Source: Front Cell Infect Microbiol. 2022 Oct 13;12:1006117. doi: 10.3389/fcimb.2022.1006117 (PMC9606567; doi:10.3389/fcimb.2022.1006117)
Supplement: Supplementary file 1 [file DataSheet_1.zip › Supplementary Material/Supplementary_Material.docx]

Supplementary Material

Supplementary Table 1. The species detected in 15 patients (20 samples) infected with *C. psittaci*.

Supplementary Table 2. The dominant species were identified among patients infected with *C. psittaci* according to the relative abundance ≥ 5% in bacteria, ≥50% in fungi, and ≥10% in virus by the mNGS analysis.

**Supplementary Table 3.** Suspected coinfection analysis by the mNGS analysis results.

**Supplementary Table 4.** The susceptibility test results of patients with culture positive.

Supplementary Table 5. The comparation of mNGS analysis results with the mNGS reports and bacterial culture results.

Supplementary Table 6. The mNGS reports identified 57 pathogens (12 pathogens of confirm,45 pathogens to suspect) from the samples.


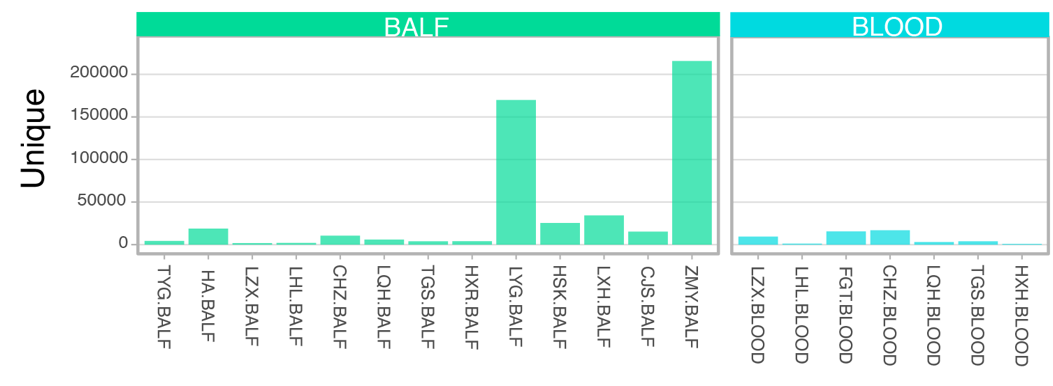


**Supplementary Figure 1.** The unique counts of BALF and blood samples of *C. psittaci* pneumonia patients to reveal the abundance of species.


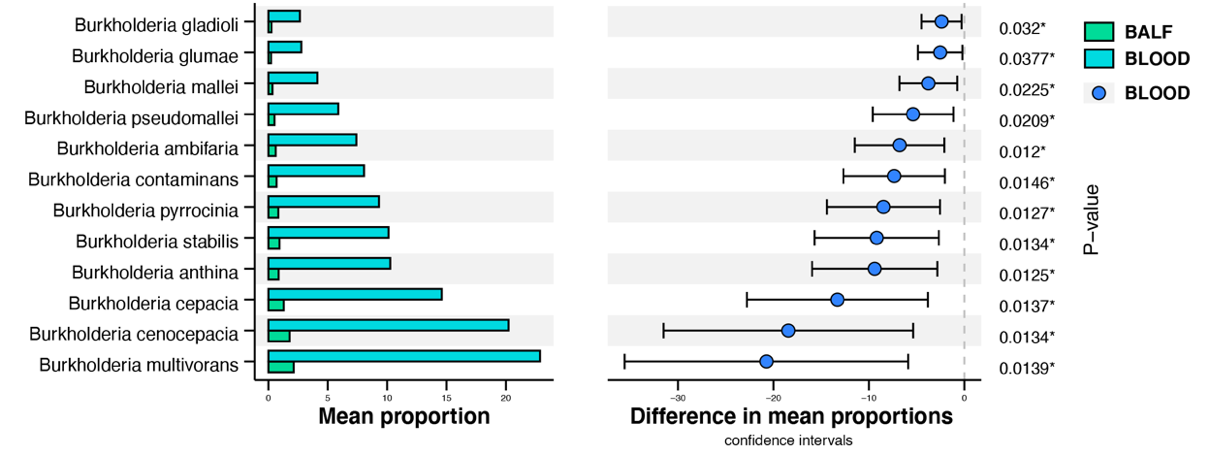


**Supplementary Figure 2.** The relative abundance of *Burkholderia* in the BALF and blood samples of *C. psittaci* pneumonia.
