# Supplementary figures and images for: Clinical diagnosis and etiology of patients with Chlamydia psittaci pneumonia based on metagenomic next-generation sequencing
Source: Front Cell Infect Microbiol. 2022 Oct 13;12:1006117. doi: 10.3389/fcimb.2022.1006117 (PMC9606567; doi:10.3389/fcimb.2022.1006117)

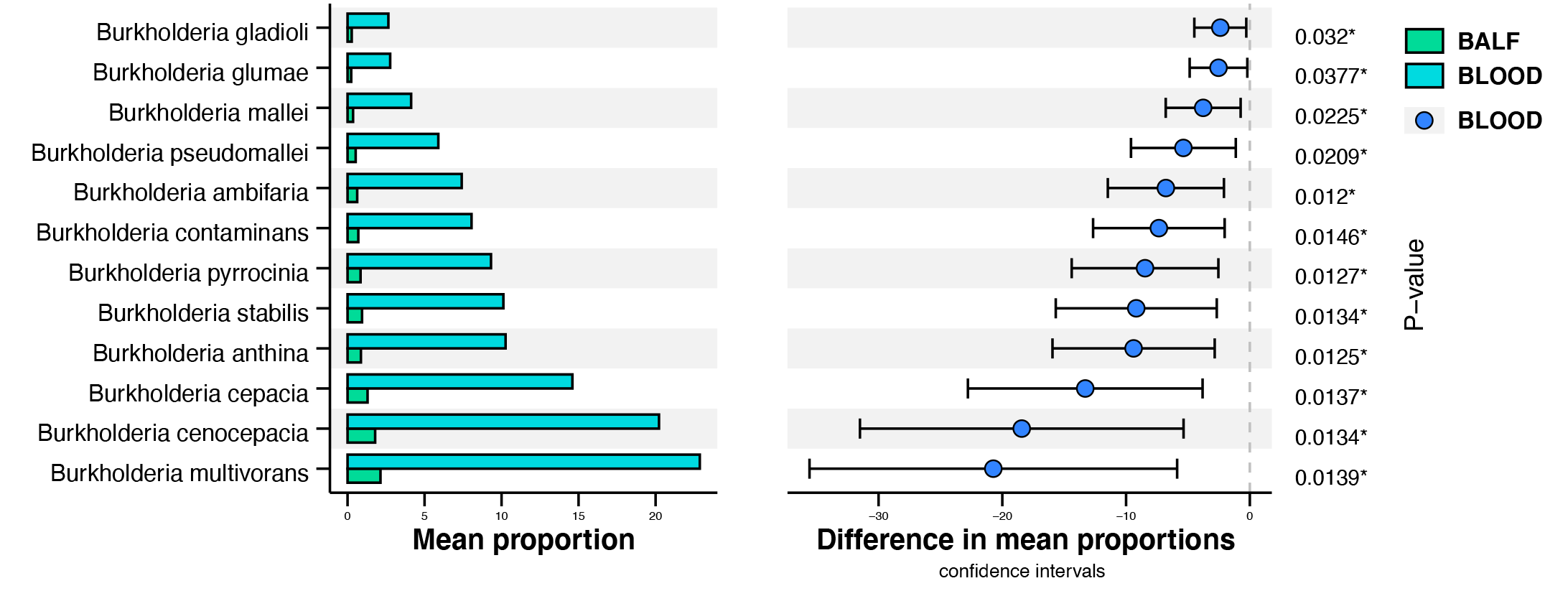

Supplement: Supplementary file 1 [file DataSheet_1.zip › Supplementary Material/supplementary figure 2.tiff]

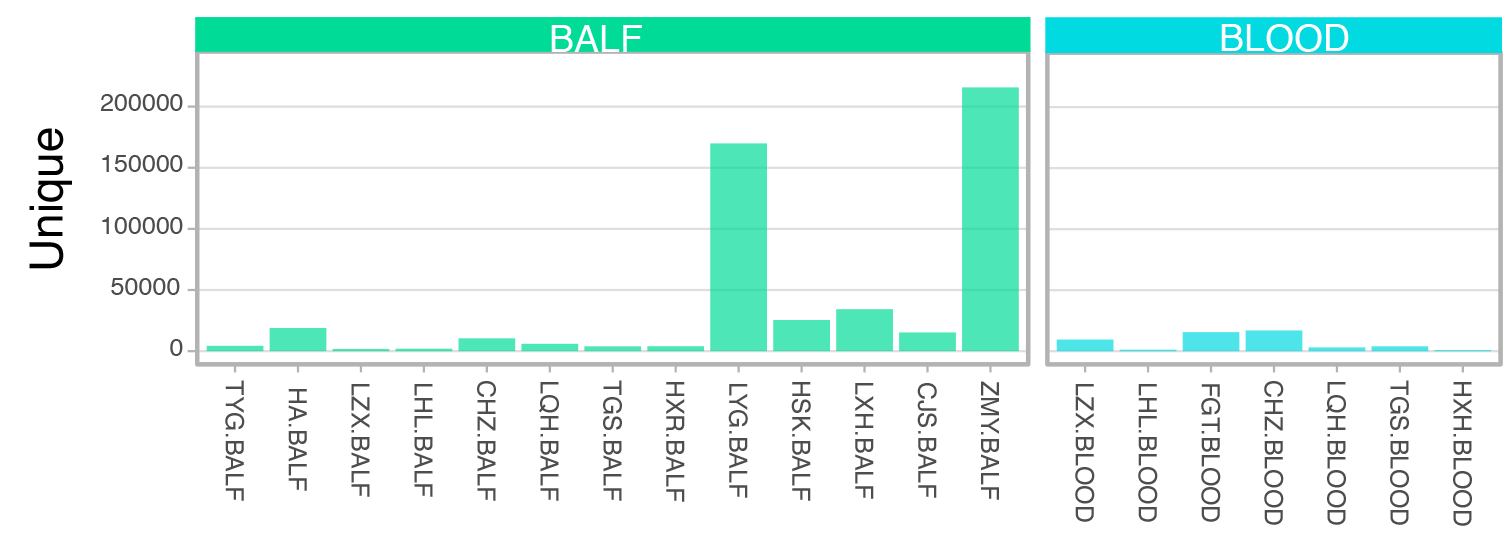

Supplement: Supplementary file 1 [file DataSheet_1.zip › Supplementary Material/supplementary figure 1.tiff]
